# Speed of leukemia development and genetic diversity in xenograft models of T cell acute lymphoblastic leukemia

**DOI:** 10.18632/oncotarget.9313

**Published:** 2016-05-12

**Authors:** Sandrine Poglio, Daniel Lewandowski, Julien Calvo, Aurélie Caye, Audrey Gros, Elodie Laharanne, Thierry Leblanc, Judith Landman-Parker, André Baruchel, Jean Soulier, Paola Ballerini, Emmanuelle Clappier, Françoise Pflumio

**Affiliations:** ^1^ Commissariat à l'Energie Atomique et aux Energies Alternatives (CEA), DSV-IRCM-SCSR-LSHL, UMR 967, Fontenay-aux-Roses, France; ^2^ INSERM, U967, Fontenay-aux-Roses, France; ^3^ Université Paris Diderot, Sorbonne Paris Cité, UMR 967, Fontenay-aux-Roses, France; ^4^ Université Paris-Sud, UMR 967, Fontenay-aux-Roses, France; ^5^ CEA, DSV-IRCM-SCSR-LRTS, UMR 967, Fontenay-aux-Roses, France; ^6^ Université Paris Diderot, Paris, France; ^7^ Assistance Publique-Hôpitaux de Paris (AP-HP), Département de Génétique, UF de Génétique Moléculaire, Hôpital Robert Debré Paris, France; ^8^ INSERM, UMR1053 Bordeaux Research in Translational Oncology (BaRITOn),Bordeaux, France; ^9^ Université de Bordeaux, Bordeaux, France; ^10^ AP-HP, Service d'hématologie Pédiatrique, Hôpital Robert Debré, Paris, France; ^11^ AP-HP, Service d'hématologie Pédiatrique, Hôpital Armand Trousseau, Paris, France; ^12^ AP-HP, Laboratoire d'Hématologie, Hôpital Saint-Louis, Paris, France; ^13^ Team Genome and Cancer, U944 INSERM, Paris, France

**Keywords:** T-ALL, leukemia initiating cells, clonal selection, CD34, xenograft

## Abstract

T cell acute lymphoblastic leukemia (T-ALL) develops through accumulation of multiple genomic alterations within T-cell progenitors resulting in clonal heterogeneity among leukemic cells. Human T-ALL xeno-transplantation in immunodeficient mice is a gold standard approach to study leukemia biology and we recently uncovered that the leukemia development is more or less rapid depending on T-ALL sample. The resulting human leukemia may arise through genetic selection and we previously showed that human T-ALL development in immune-deficient mice is significantly enhanced upon CD7^+^/CD34^+^ leukemic cell transplantations. Here we investigated the genetic characteristics of CD7^+^/CD34^+^ and CD7^+^/CD34^−^ cells from newly diagnosed human T-ALL and correlated it to the speed of leukemia development. We observed that CD7^+^/CD34^+^ or CD7^+^/CD34^−^ T-ALL cells that promote leukemia within a short-time period are genetically similar, as well as xenograft-derived leukemia resulting from both cell fractions. In the case of delayed T-ALL growth CD7^+^/CD34^+^ or CD7^+^/CD34^−^ cells were either genetically diverse, the resulting xenograft leukemia arising from different but branched subclones present in the original sample, or similar, indicating decreased fitness to mouse micro-environment. Altogether, our work provides new information relating the speed of leukemia development in xenografts to the genetic diversity of T-ALL cell compartments.

## INTRODUCTION

Hematopoiesis is the normal process for whole mature blood cell production. This process takes place in the bone marrow (BM) and its deregulation is the basis of multiple malignancies, including leukemia. Development of leukemia occurs through progressive cell transformations in early immature hematopoietic stem or progenitor cells, such as in chronic (CML) and acute myeloid leukemia (AML) (reviewed in [1]). Careful analyses of leukemic subpopulations led to postulate of the presence of leukemia initiating/stem cells that represent an ideal target for anti-cancer therapies [2].

T cell acute lymphoblastic leukemia (T-ALL) result from an aberrant accumulation of immature T cells bearing multiple oncogenic lesions in blood and BM [3]. Access to patient samples helped developing relevant experimental models to study this hematologic disorder [4]. Using xenograft models, several groups tested whether, as for AML [5, 6] T-ALL include subpopulations of cells that are more fitted to serial re-initiation of leukemic development following transplantation into immunodeficient mice [4, 7, 8]. Such cells were thereafter found enriched amongst cells expressing or not CD34, CD1a, CD4 and/or CD7 surface markers [7–9]. In this context our group showed that CD7^+^/CD34^+^ cells sorted from several T-ALL samples promote T-ALL development more frequently and faster than their CD7^+^/CD34^−^ counterparts [9]. However, depending on the considered leukemia sample and contrary to CD7^+^/CD34^+^ cells found in normal thymocyte differentiation, we observed that T-ALL-derived CD7^+^/CD34^+^ cells are not uniformly early T cell progenitors as they may co-express CD34 with mature surface markers such as CD4, CD8 and/or CD3/TCRαβ [9]. The reason why CD7^+^/CD34^+^ leukemic cells are more aggressive is thus not uniformly related to their immaturity and remains obscure in many cases. CD34 is a glycoprotein promoting normal T-cell adhesion through L-selectin interaction with endothelial cells [10], which suggests that functional differences between CD34^−/+^ cell fractions may be explained by distinct adhesion and migration inside the bone marrow (BM). Beside we have shown that leukemia development following transplantation of T-ALL patient cells into immunodeficient mice coincides with selection of genetic subclones that bear an enhanced ability to re-initiate T-ALL[11] and leukemia development is more or less rapid correlating with several biological and molecular characteristics of leukemia samples [12].

On the follow up of those studies we genetically characterized CD7^+^/CD34^+^ and CD7^+^/CD34^−^ T-ALL cells to understand the biological basis of their distinct functional properties. We also correlated these characteristics with the speed of leukemia development in xenografts. We observed that CD7^+^/CD34^+/−^ cell fractions from the T-ALL we studied endowed with fast promoting leukemia activity are homogenous in terms of genetics and migration/niche adhesion abilities. Besides CD7^+^/CD34^+/−^ cell fractions from studied T-ALL samples with delayed leukemia development activity are either genetically heterogeneous or homogenous. In such slow developing T-ALL differential CD34 expression may thus distinguish genetically diverse cell fractions with distinct abilities to re-establish leukemia in mice or genetically homogenous cell fractions with a weak growing fitness in mouse. These results uncover the broad diversity between leukemia samples and between leukemic sub-fractions in their functional ability to develop in xenograft models. They provide new information to understand T-ALL development in xenografts, relating phenotypic cell sub fractionating, genetic diversity and speed of leukemia development.

## RESULTS

### CD34+/− cells from fast growing samples show identical genomic alterations and phenotypes

**Figure 1:**
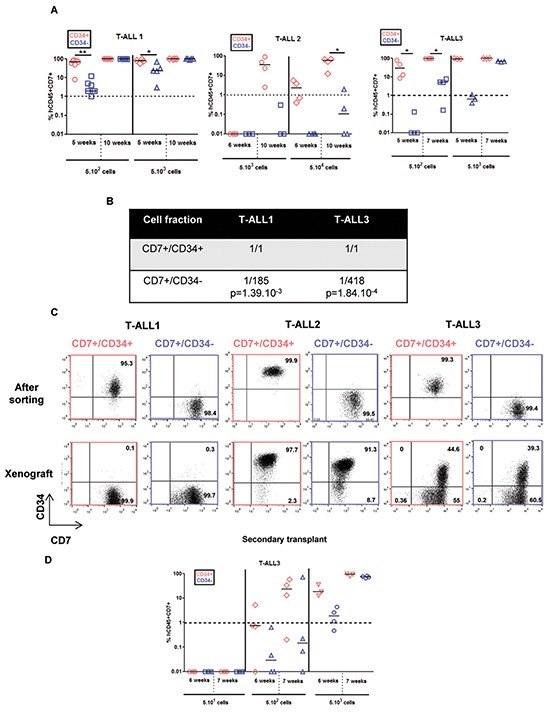
CD7^+^/CD34^+^ and CD7^+^/CD34^−^ cell fractions from 3 fast growing T-ALL samples have different kinetics of leukemia development but leukemic cells derived from xenograft harbouring same phenotype 5×102 (T-ALL1, T-ALL3), 5×103 (T-ALL1, T-ALL2, T-ALL3), 5×104 (T-ALL2) of CD7^+^/CD34^+^ (red points) and CD7^+^/CD34^−^ (blue points) T-ALL cells/mouse were injected by iv route into NSG mice. **A.** Engraftment kinetics for individual mice. The percent of hCD45^+^hCD7^+^ leukemic cells detected by FACS in BM samplings or at euthanasia (end points) are shown. Statistics were determined using the 2-tailed Mann and Whitney test.*p<0.05. **B.** Frequency of cells endowed with leukemia initiation ability in different patient samples was determined using Extreme Limiting Dilution analysis software (http://bioinf.wehi.edu.au/software/elda/) using 3 cell doses of T-ALL1 and T-ALL 3 (5×101, 5×102 and 5×103cells /mouse). **C.** CD34 and CD7 expression in leukemic cells following cell sorting from newly diagnosed samples (upper panel) and from human hCD45^+^hCD7^+^ cells recovered from BM of xenografted NSG mice (lower panel). CD34 positivity was set according to isotype controls. **D.** Leukemia development following secondary transplants of total BM cells isolated from primary mice transplanted with CD7^+^/CD34^+^ (red) or CD7^+^/CD34^−^ (blue) sorted T-ALL cells. Results are from T-ALL3.

Different numbers (5×10^2^ to 5×10^5^ cells/mouse) of CD7^+^/CD34^+^ and CD7^+^/CD34^−^ cells sorted from the blood of newly diagnosed human T-ALL (T-ALL1-6, Supplementary Table S1) were transplanted into NOD/SCID/γc^−/−^ (NSG) mice based on previous results obtained with these samples [9, 12]. BM cells were sampled regularly from 5 to 20 weeks and leukemia development was measured *via* the percentage of hCD7^+^CD45^+^ cells [4]. Leukemia development ability was quantified using the proportion of NSG mice with over 1% hCD7^+^CD45^+^ cells at a given time point (5 weeks for T-ALL1, 7 weeks for T-ALL3 and 20 weeks for T-ALL5). The time to leukemia (TTL) development was variable in the different T-ALL cases and T-ALL1-3 leukemia developed as early as 5-6 weeks after injection upon 5-50×10^2^ cells (Figure 1A) all three being thus considered as short TTL [12]. In accordance with [9], CD7^+^/CD34^+^ cells were more prone to generate leukemia than CD7^+^/CD34^−^ cells in the studied T-ALL cases, albeit this difference could be reduced as in T-ALL1 (Figure 1A–1B). For T-ALL3 case, cells isolated from primary mice re-initiated leukemia with a slight delay for CD7^+^/CD34^−^ cells compared to CD7^+^/CD34^+^ cells in secondary recipient (Figure 1D).

**Figure 2:**
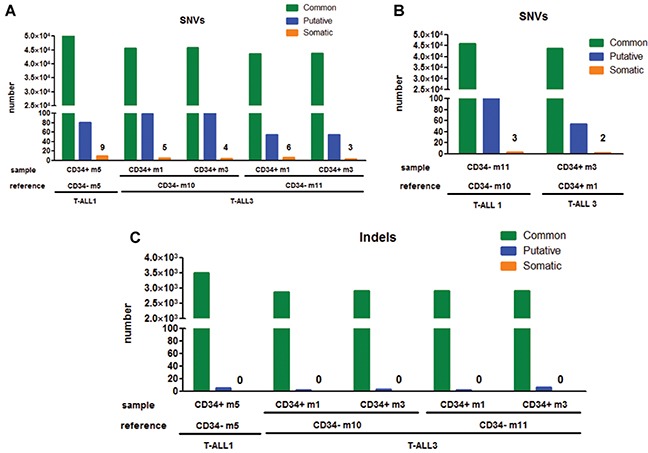
Comparative analyses of genomic alterations identified in CD7^+^/CD34^+^ vs CD7^+^/CD34^−^ derived xenograft leukemia **A–B.** For T-ALL1 and 3, we performed whole-exome sequencing on xenografted CD7^+^/CD34^+^ and matched xenografted CD7^+^/CD34^−^ cells DNA (A) or by comparing the data of CD7^+^/CD34^+^ and CD7^+^/CD34^−^ cells together (B). Number of Single Nucleotide Variants (SNVs) was defined as mentioned in Supplementary Material and Methods. Number of alterations were detailed for shared variations (Common), hazardous alterations (Putative) and differences in tested sample vs reference (Somatic). **C.** Number of small insertion or deletion (Indels) was shown for xenografted CD7^+^/CD34^+^ and matched xenografted CD7^+^/CD34^−^ cells DNA as described above.

As leukemia development relies on clonal selection in xenograft [11], we hypothesized that the difference in aggressiveness between CD7^+^/CD34^+^ and CD7^+^/CD34^−^ cells relates on the presence in both sub-fractions of distinct genetic subclones. Genomic alterations being very frequent oncogenic alterations in T-ALL [3], array-CGH analyses were performed in order to investigate whether molecular lesions would segregate with the distinct cell populations at diagnosis and at what extent they would be detected after xenograft. For T-ALL1-3 cases, sorted CD7^+^/CD34^+/−^ populations at diagnosis, as well as cells recovered from engrafted mice, showed identical genetic alterations with no evidence of major clonal selection during leukemia development in xenograft (Supplementary Tables S2, S3 and S4). These results were confirmed using whole-exome sequencing (WES) of DNA from xenografted CD7^+^/CD34^+^ cells and matched CD7^+^/CD34^−^ cells in T-ALL1 and T-ALL3. This analysis yielded a mean depth of 115-141x and 88-90% of targeted bases were covered to a depth of 25× or more. Comparison of CD7^+^/CD34^+^-derived and CD7^+^/CD34^−^ derived xenografted cells identified very few (3 to 9) somatic Single Nucleotide Variants (SNVs) and no small insertion or deletion (indel) (Figure 2A, 2C). Similar results were obtained by comparing the data of CD7^+^/CD34^+^ and CD7^+^/CD34^−^ cells intrinsically but from different mice (Figure 2B), indicating differences between CD34^+^ and CD34^−^ derived xenografted cells could be related to mouse rather than to injected cell fraction differences. Importantly no alteration linked to CD34 expression and no high functional consequences of somatic variants was predicted by Variant Effect Predictor software. Leukemic cells recovered from mice transplanted with CD7^+^/CD34^+^ or CD7^+^/CD34^−^ cells from T-ALL1-3 were also phenotypically identical in terms of CD34/CD7 and CD4/CD8 marker expression further supporting the similarity of the xenografts issued from both cell fractions (Figure 1C and Supplementary Figure S1B). Interestingly differences existed between xenografted cells and the original sample (Figure 1C and Supplementary Figure S1), suggesting a change in surface marker expression levels maybe in relation with the mouse microenvironment. Altogether these results indicated that the genetic clonal architecture is rather simple in cases of fast growing T-ALL with no major genetic differences between CD34^+^ and CD34^−^ cells.

### CD34 expression does not promote enhanced cell homing in fast growing leukemic cells

**Figure 3:**
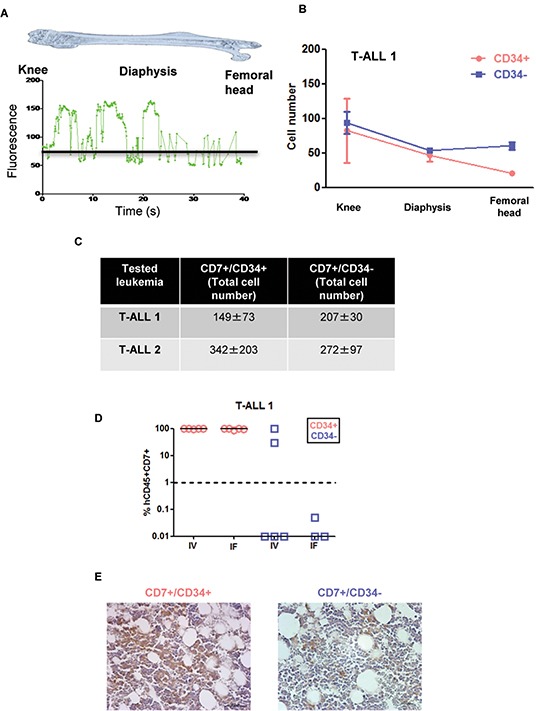
Leukemia development obtained from CD7^+^/CD34^+^ and CD7^+^/CD34^−^ cell subfractions do not rely on distinct early homing properties **A.** CD7^+^/CD34^+^ or CD7^+^/CD34^−^ cells (5×104/mouse) from T-ALL1 and T-ALL2 were labelled with CFSE and injected intravenously into NSG mice. Shown is the mean of the relative fluorescence units detected throughout the femur by the optical fibers per individual video frame. The black line represents the background level **B.** Quantification of the images obtained 72 hours after transplantation. Results represent manually counted cell numbers (mean +/− SD) found on 200 video frames for each femur section (the knee, the diaphysis, and the femoral head). A total of 4 mice were studied and 2 femurs for each mouse injected with T-ALL1 subfractions were analysed in this experiment. **C.** Total leukemic cell number in 2 femurs 72h after CD7^+^/CD34^+^ or CD7^+^/CD34^−^ cell transplantation. Results are the mean +/− SD of 2 and 4 mice for T-ALL1 and 2 respectively. **D.** CD7^+^/CD34^+^ or CD7^+^/CD34^−^ T-ALL1 (5×101/mouse) cells were injected by intravenous (IV) or intrafemoral (IF) routes in NSG mice. Percent of hCD45^+^/CD7^+^ leukemic cells was evaluated in BM 9 weeks after transplant of T-ALL1. **E.** Immunohistochemistry of BM of mice transplanted with 5×104 CD7^+^/CD34^+^ or CD7^+^/CD34^−^ T-ALL1 cells. The analysis was performed 3 weeks after transplant. Shown in brown color is the labelling of human leukemic cells with a specific anti-human CD45 antibody.

As CD34 regulates cell migration and adhesion [10], we studied whether the distinct leukemia development activity between CD34^+^ and CD34^−^ cells from T-ALL1 and T-ALL2 was based on a differential homing capacity. Sorted CD7^+^/CD34^+^ and CD7^+^/CD34^−^ cells were stained with Carboxyfluorescein Diacetate Succinimidyl Ester (CFSE) before transplantation and tracked using a Cell Vizio-instrument (Mauna Kea Technologies) to monitor their migration and adhesion to the niches into BM as developed in [13] (Figure 3A and Supplementary Figure S2). Leukemic CFSE^+^ cells were detectable only 72h after injection, with no difference with respect to the engrafting cell number or invaded BM areas between CD7^+^/CD34^+^ and CD7^+^/CD34^−^ cells (Figure 3A–3C and Supplementary Figure S2B). Moreover injecting CD7^+^/CD34^+^ and CD7^+^/CD34^−^ cells from T-ALL1 and T-ALL2 using *iv* or intra-femur route did not change the difference of engraftment observed with both fractions (Figure 3D and Supplementary Figure S2C). Kinetic analysis of CD7^+^/CD34^+/−^ leukemic cell growth *in vivo* using immunohistochemistry did not allow early cell infiltration analysis but revealed higher late infiltration level for CD7^+^/CD34^+^-derived xenografts compared to CD7^+^/CD34^−^-derived leukemia (Figure 3E) in accordance with flow cytometry datas (Figure 1A). Thus differences in leukemia development abilities between CD7^+^/CD34^+^ and CD7^+^/CD34^−^ cells in fast growing T-ALL do not rely on distinct early homing properties.

### T-ALL with delayed leukemia development ability are either genetically heterogeneous or homogenous and CD34 expression may discriminate specific subclones

**Figure 4:**
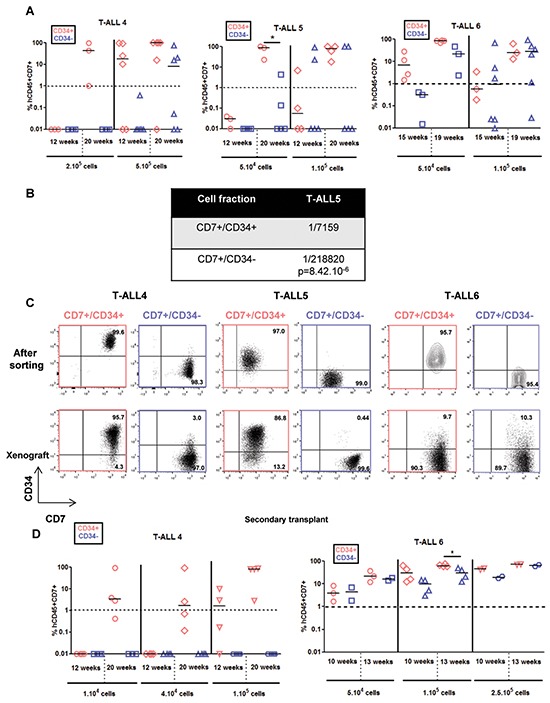
CD7^+^/CD34^+^ and CD7^+^/CD34^−^ cell fractions from slow growing T-ALL samples generate leukemia with distinct kinetics and phenotypes 5×104 (T-ALL5 and T-ALL6), 1-2×105 (T-ALL4-6) and 5×105 (T-ALL4) of CD7^+^/CD34^+^ (red) and CD7^+^/CD34^−^ (blue) T-ALL cells/mouse were injected by iv route into NSG mice. **A.** Engraftment levels for individual mice. Shown are the percent of hCD45^+^hCD7^+^ leukemic cells detected by FACS in BM samplings or at euthanasia. **B.** Leukemic cell development ability quantified as in Figure 1B. 3 cell doses of T-ALL5 (1×104, 5×104 and 1×105cells /mouse) were tested to obtain the indicated frequency. **C.** CD34 and CD7 expression in T-ALL4-6 leukemic cells from newly diagnosed samples (upper panel, cell sorted fractions) and from human hCD45^+^hCD7^+^ cells recovered from BM of xenografted NSG mice (lower panel). CD34 positivity was set according to isotype controls. **D.** Leukemia development of secondary transplants of total BM of primary mice engrafted with CD7^+^/CD34^+^ or CD7^+^/CD34^−^ cells from T-ALL4 and T-ALL6 cells. Shown are the percent of human hCD45^+^hCD7^+^ leukemic cells at defined sampling time, the last time being at sacrifice. Statistics were determined using the 2-tailed Mann and Whitney test.*p<0.05.

**Figure 5:**
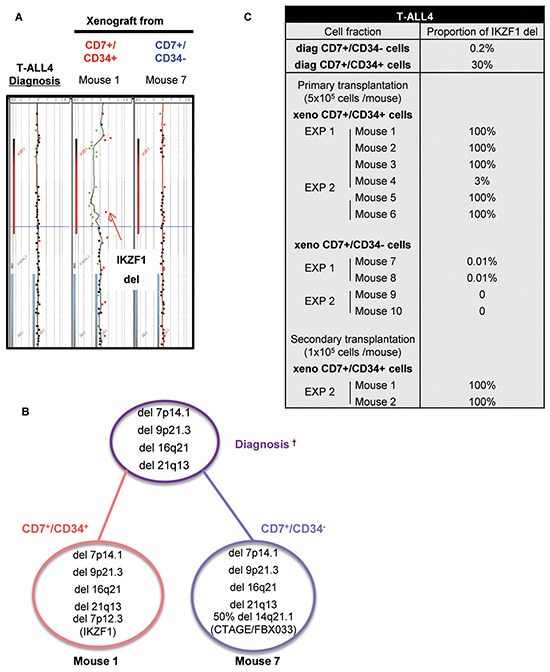
CD34 expression unveils leukemic cells with genetic differences and different aggressiveness *in vivo* **A.** Comparative Genomic Hybridization (CGH) analysis of CD7^+^/CD34^+^ and CD7^+^/CD34^−^ cells at diagnosis and from total human cells recovered after xenograft of CD7^+^/CD34^+^ and CD7^+^/CD34^−^ cells from T-ALL4 patients. Shown is the profile of the IKZF1 deletion in CD7^+^/CD34^+/−^-derived xenograft leukemia compared to diagnosis. **B.** Summary of array-CGH results obtained at diagnosis and after xenograft of human CD7^+^/CD34^+^ and CD7^+^/CD34^−^ cells. Percentages of cells with genetic alterations were evaluated based on log2 ratio values.† are shown only genomic alteration in cells detected by array-CGH. Red and blue circles indicate genomic alterations in xenograft derived CD7^+^/CD34^+^ and CD7^+^/CD34^−^ cells respectively. Purple circle shown genomic alterations at diagnosis. **C.** Quantitative analysis of IKZF1 deletions in CD7^+^/CD34^+^- and CD7^+^/CD34^−^ cell fractions at diagnosis and after primary/secondary xenografts (≥2 mice/experiment) in 2 independent experiments (EXP 1 and 2). The table summarizes the proportion of human IKZF1 deleted cells determined by breakpoint-specific quantitative PCR on total DNA.

We also studied 3 T-ALL that have a delayed leukemia development capacity [12]. T-ALL4-6 required 12 to 20 weeks to develop in mice even though >10 times more cells were transplanted from either CD7^+^/CD34^+^ or CD7^+^/CD34^−^ cell sub-fraction (Figure 4A). As for T-ALL1-3, CD34^+^ cells produced leukemia in xenograft faster meaning they were more aggressive than CD34^−^ cells although increasing the injected cell number allowed to observe and quantify leukemia development also in CD7^+^/CD34^−^ cells of all 3 slow-growing T-ALL (Figure 4A–4B). Interestingly except for T-ALL6, the expression of CD4 and CD8 cell surface markers were distinct between xenografts derived from CD7^+^/CD34^+^ and CD7^+^/CD34^−^ cells (Supplementary Figure S1B) although, within the same sample, CD34 and CD7 expression remained similar to the diagnosis sub-populations from which they derived (Figure 4C). The differences observed at diagnosis in terms of CD4 and CD8 expression between CD34^+^ and CD34^−^ cells were accentuated after xenografts except again for T-ALL6 (Supplementary Figure S1). Array-CGH analyses revealed that leukemic cells recovered from mice transplanted with CD7^+^/CD34^+^ or CD7^+^/CD34^−^ cells from T-ALL4 and T-ALL5 shared several molecular lesions with the cells at diagnosis from which they derived, indicating clonal relationship (Figure 5A–5B and Supplementary Figure S3). However cells from T-ALL4 and T-ALL5 xenografts carried additional distinct genetic lesions (Figure 5A–5B and Supplementary Figure S3), indicating clonal evolution during leukemia development in mice. In the case of T-ALL4, CD7^+^/CD34^+^ derived-xenografts displayed a novel short genomic deletion of IKZF1 on the chromosome 7p12.3 not detected by array-CGH in cells at diagnosis (IKZF1del, Figure 5A–5B). IKZF1 is a tumour suppressor gene whose loss of function drives NOTCH activation and promotes T-ALL in mice [14] and which is altered recurrently in B-ALL [15], and sometimes in T-ALL [16]. IKZF1del was neither detectable by CGH-array in sorted CD7^+^/CD34^−^ cells and was not selected upon transplant of these cells in NSG mice. Indeed it was absent from CD7^+^/CD34^−^-derived xenografts that carried a chromosome 14q21 deletion in half of cells (Figure 5B). These results indicated that T-ALL4 CD7^+^/CD34^+^ and CD7^+^/CD34^−^-derived xenografts originated from distinct but related genetic subclones that were distinctly represented in CD34^+/−^ cell fractions. By using quantitative breakpoint-specific PCR to perform clonal tracking of IKZF1del [17] in fractions sorted at diagnosis and in their derived xenografts, we detected IKZF1del in 30% of CD7^+^/CD34^+^ cells at diagnosis, as compared to 0.2% of CD7^+^/CD34^−^ cells. In CD7^+^/CD34^+^-derived leukemia, the proportion of IKZF1del positive cells raised to 100% in virtually all (5^+^/6 mice, 2 experiments) mice, indicating the reproducible selection of the IKZF1del clone, while it was barely detectable in CD7^+^/CD34^−^-derived xenografts (0.01% in 2/4 mice, 2 experiments, Figure 5C). Interestingly only xenografts from CD7^+^/CD34^+^ sorted T-ALL4 leukemic cells were able to initiate leukemia in secondary transplants (Figure 4D) in accordance with our previous results [9] and secondary T-ALL were 100% IKZF1del positive cells (Figure 5C). As expected from [11], these primary xenografted cells were more aggressive than diagnostic cells as secondary transplant of 10^5^ cells from CD7^+^/CD34^+^ xenografts generated leukemia as early as 12 weeks post-transplant compared to no leukemia development at the same time point when two times more sorted CD7^+^/CD34^+^ diagnostic cells had been transplanted (Figure 4A). These results suggested that enrichment of IKZF1del subclone participates into the strong CD7^+^/CD34^+^ leukemia initiation ability in NSG mice. In the case of T-ALL5, two major genetically related sub-clonal populations were detected in the diagnosis sample, sharing identical CDKN2A/B and TCRA/D rearrangements but harbouring distinct chromosome 6q deletions (51Mb and 45Mb), each in approximately half of leukemic cells (Supplementary Figure S3A). T-ALL5 xenografts originating from CD7^+^/CD34^+^ and CD7^+^/CD34^−^ sorted cells harboured mainly one of these 6q deletions, either 6q/45Mb or 6q/51Mb, respectively (Supplementary Figure S3B–S3C), a minor 6q/43.2Mb deletion being also detected in mouse 3 injected with CD7^+^/CD34^+^ cells, suggesting that CD7^+^/CD34^+^ and CD7^+^/CD34^−^ cells at diagnosis had probably distinct 6q deletions, a result we could not verify due to patient material limitation. Furthermore, in 2/3 mice, xenografts derived from T-ALL5 CD7^+^/CD34^+^ cells harboured additional genetic lesions, indicating that the original CD7^+^/CD34^+^ fraction contained multiple minor subclones (Supplementary Figure S3B–S3C). In the case of T-ALL6, sorted CD7^+^/CD34^+/−^ derived cells recovered from engrafted mice showed identical genetic alterations (Supplementary Table S5) suggesting that long-term developing T-ALLs may as well be genetically homogenous. As expected, T-ALL6 cells derived from primary recipient re-initiated leukemia in secondary faster than primary transplant with a slight delay for CD7^+^/CD34^−^ cells compared to CD7^+^/CD34^+^ cells (Figure 4D).

These results show that long-term developing T-ALL may be more heterogeneous than short-term engrafting T-ALL although this is not a general rule. Multiple subclones were found in 2/3 samples unequally distributed within the CD7^+^/CD34^+^ and CD7^+^/CD34^−^ fractions, and their respective frequencies as well as oncogenic events seem to play roles in the specific abilities of CD34^+/−^ cells to perpetrate leukemia in xenograft models.

## DISCUSSION

Altogether our results suggest that T-ALL that mediate rapid leukemia development in immunodeficient mice are genetically simple whereas T-ALL taking a longer time to re-initiated leukemia may be genetically more diverse, although genetically homogenous T-ALL can also be detected in this group. Indeed we have found that 3/3 tested short term engrafting T-ALLs contain a predominant genetically defined clone endowed with xenograft leukemia development capacity that segregates with CD7^+^/CD34^+^ and CD7^+^/CD34^−^ cell fractions, although the latter fraction was poorer in such function. In these T-ALL the clonal selection process has probably taken place in patients and the selected molecular mechanisms support leukemia re-establishment in NSG mice. In fact, our work focused on the 60% of paediatric T-ALL that engraft into NSG mice. It is likely that, among non engrafting T-ALL samples there are genomically homogeneous samples whose cell growth are not permissive to mouse microenvironment. In agreement, we observed that one of the 3 samples with long term engrafting potential was also genetically simple indicating that the gene program supporting leukemia development in this patient did not provide strong signals fitting the mouse environment. In this case a minor cross contamination between cell fractions at sorting leading to this result was excluded when CGH-array, original and xenografts phenotypes analyses and leukemia development kinetics were considered altogether.

We recently documented the fact that fast leukemia development in NSG mice does not significantly correlate with bad prognosis in patients [12]. Putting both results together may imply that current treatments are more efficient on genetically homogenous leukemia, making sense with the fact that relapse generally develops as new leukemia originating from related or unrelated minor subclones [18, 19]. Testing additional samples in relation to their genetics will help answer this question.

Because of the genetic similarities found in the fast engrafting T-ALL, we investigated whether CD34 expression is associated with enhanced leukemia development because of its implication in migration/adhesion [10]. We tested the homing of CD7^+^/CD34^+^ and CD7^+^/CD34^−^ cells after transplant into NSG mice but found no relation between CD34 expression and migration properties or BM niching. As array-CGH only detects genomic unbalances, we cannot exclude that other discrete genetic alterations or differential epigenetic states may discriminate CD7^+^/CD34^+^ and CD7^+^/CD34^−^ cell fractions in these rapidly engrafting T-ALL albeit WES analyses of fast engrafting samples confirmed the genetic proximity of those cell fractions. Because we recently described that fast engrafting T-ALL exhibit enhanced NF-κB pathway activation [12], it might be interesting to look into this activation in the CD7^+^/CD34^+^ and CD7^+^/CD34^−^ sub-fractions, also because NF-κB pathway is activated following extracellular signals, such as those provided by inflammation, and we have previously shown that the pro-inflammatory cytokine IL-18, that activates NF-κB, participates into T-ALL development [20].

By contrast, 2/3 tested slow engrafting T-ALL were detected to be genetically diverse compared to rapid engrafting T-ALL. In those cases, CD7^+^/CD34^+/−^ cell fractions gave rise to genetically and phenotypically different leukemia in NSG mice. Even though NSG mice are severely immune deficient and thus may allow any leukemic clone to grow, transplantation of multiclonal T-ALL cell fractions, such as those sorted from slow developing T-ALL, results in clonal selection that reduce the intrinsic complexity of T-ALL development and reveal the propensity of certain genetic events to support leukemia development in mice. These results are supported by recent findings showing that NSG mice used as recipients for AML patient blood samples were found to select particular genetic subclones with distinct cell surface phenotypes [21]. Interestingly, starting with the same patient but using different mouse recipients, i.e. NSG mice producing human interleukin 3, stem cell factor and GM-CSF previously described as better recipients for human AML [22], the same authors observed the reproducible selection of different subclones, indicating the important role of the mouse microenvironment in the selection process, at least for myeloid leukemia [21]. Such results would be interesting to document in the case of T-ALL.

Mouse microenvironment may modify T-ALL engraftment ability notably after primary xenograft, independent of clonal genetic selection. In T-ALL3 case, the majority of CD7^+^/CD34^+/−^ derived-cells isolated from BM of primary mice are CD34^−^ and re-initiate leukemia in secondary recipient only with a slight delay for CD7^+^/CD34^−^ cells compared to CD7^+^/CD34^+^ cells. This result suggests that CD34 expression is associated with enhanced leukemia development only at diagnosis and not after primary xenograft. This further highlights the relevance to work with fresh patient samples devoid of mouse microenvironment imprinting.

Interestingly CD7^+^/CD34^+^ cells of the slow developing T-ALL4 contained a genetic subclone recurrently selected in xenografts that harbours a deletion of the tumour suppressor IKAROS. Loss of IKAROS function is a driver of Notch pathway activation capable to induce T-ALL in mouse and infrequently mutated in patients [14, 16]. This subclone was well-represented (30%) in the CD7^+^/CD34^+^ cell fraction at diagnosis whereas it was very infrequent (0.2%) among CD7^+^/CD34^−^ cells. It is likely that this high frequency and maybe the activation of Notch pathway [4] contributed to the selection of this subclone and subsequent secondary transplantation maintained this clone representing 100% of the cells from most primary and secondary grafts. Xenografts from CD7^+^/CD34^−^ cells did not select this rare subclone but other ones, in particular bearing a deletion of CTAGE5, a T-lymphoma tumour antigen associated with endoplasmic reticulum that mediate collagen secretion [23]. Interestingly enough, these CD7^+^/CD34^−^ derived xenografts developed slowly and were depleted in leukemia propagating activity in secondary transplants, outlining the more aggressive behaviour of the IKAROS deleted subclone. This result may suggest that as in breast cancer [24], subclones interactions participate into tumour growth and the lack of CD7^+^/CD34^+^ imprinting on CD7^+^/CD34^−^ cells may impair the ability of CD7^+^/CD34^−^ cells to engraft in secondary recipients.

Overall, our work reveals that T-ALL cell subpopulations isolated based on cell surface markers, such as here CD34 and CD7 expression, and bearing differential leukemia initiating activity may contain distinct clones, with genetic and probably also, but yet not depicted, epigenetic differences. In the most genetically diverse T-ALL xenograft, leukemia development is slow most likely in relation with the frequency of cells with the best fitness to the mouse microenvironment. In this idea, genetically homogenous T-ALL engraft either rapidly or very slowly. As we only studied engrafting samples we cannot exclude that non-engrafting samples could escape this rule. Understanding why those samples do not engraft may help resolve this question and also may improve long term samples engraftment, leading to patient faithful models for testing novel T-ALL therapies.

In general this study allows to better understand leukemia development and heterogeneity when using xenograft models for T-ALL studies. This work also clearly outlines the relation between clonal selection/diversity and T-ALL leukemia initiating concept in T-ALL.

## MATERIALS AND METHODS

### Patients

Blood samples from 6 pediatric or young adult T-ALL patients were collected at diagnosis in pediatric hematological departments from hospital Armand Trousseau and hospital Robert Debré (Paris, France) (Supplementary Table S1). Parents or representatives of pediatric patients gave informed consent in accordance with the declaration of Helsinki and national ethic rules. Patients’ characteristics are described in Supplementary Table S1. The institutional review board (IRB00003888) of INSERM approved the manipulations of T-ALL samples (project 13-105-2).

### Processing of human T-ALL samples

All experiments were performed with primary newly diagnosed samples unless mentioned. Peripheral blood mononuclear cells (PBMC) were isolated by Ficoll centrifugation, immuno-phenotyping was done and cells were used directly or frozen in fetal calf serum (FCS) containing 10% DMSO. T cells were characterized by lymphoid CD7 cell surface expression and normal mature contaminating T-cells were excluded by Flow Activating Cells Sorting (FACS) based on their expression of sCD3^+^(hi) TCRαβ^+^(hi) CD4^+^(hi) or CD8^+^(hi) markers. CD7^+^/CD34^+/−^ cells were enriched using an INFLUX cell sorter (BD Bioscience, Le Pont de Claix, France). Sorted cells were pelleted for DNA extraction or used for xenograft experiments.

### Flow cytometry

Characterization of xenografted T-ALL samples was performed by flow cytometry. Cells were stained with fluorescein (FITC-), phycoerythrin (PE-), PE-cyanin 7 (PC7-) and allophycocyanin (APC-) conjugated mouse anti-human monoclonal antibodies specific for CD7, CD34, CD4 and CD8 (Beckman Coulter, Villepinte, France). Cell surface marker expression were analyzed on a FacsCalibur (BD Biosciences) using FlowJo software (Tree Star Inc., Ashland, OR, USA).

### Mice

NOD.Cg-Prkdc(scid)Il2rg(tm1Wjll)/SzJ (abbreviated NOD/scid/IL-2Rγ null; NSG; from The Jackson Laboratory) were irradiated with 3 Grays (IBL 637 CisBio International, France; dose rate 0.61 Gy/min) and anesthetized with isoflurane before injection of T-ALL cells [9]. CD7^+^/CD34^+/−^ leukemic cells from primary newly diagnosed T-ALL samples were injected intravenously (*iv*) through the retro-orbital sinus or in femur in 3 or more NSG mice. To monitor leukemic engraftment, BM samplings were performed after anesthesia (7.5 mg/mL Ketamin and 0.05% Xylazin) and analgesia (3μg/mL buprenorphin). Human leukemic hCD7^+^/hCD45^+^ cells were detected by FACS. Mice were sacrificed when engraftment reached at least 90% or if mice showed clinical signs of illness. At sacrifice, the percentage of leukemic cells was analyzed in total BM and xenografted cells were pelleted for DNA extraction. Frequency of cells able to initiate leukemia in different patient samples was determined using Extreme Limiting Dilution analysis software (ELDA, http://bioinf.wehi.edu.au/software/elda/). Briefly, positive mice (with over 1% hCD7^+^CD45^+^ cells) number for 3 cell doses of T-ALL1/3 and T-ALL5 was determined at a given time and loaded on ELDA software. Frequency was calculated as previously described [25]. All experimental procedures were performed in compliance with French Ministry of Agriculture regulations (animal facility registration number: A920322) for animal experimentation and in accordance with the local ethical committee (Reference number 12-015).

### *In vivo* imaging

CD7^+^/CD34^+/−^ leukemic cells were labeled with CFSE (eBioscience, Santa Clara, CA, USA) according to the manufacturers’ instructions and injected in NSG mice. 24 and 72h after injection, CFSE^+^ cells were detected in the 2 femurs by fiber optic imaging as previously described [13]. Briefly, the knee of anesthetized transplanted mice was flexed and superficially pierced with a 23-gauge needle through the joint. S300 flexible microprobe (Mauna Kea Technologies) was entered and moved slowly inside the femoral cavity all the way up to the femoral head. Video data were acquired with the CellVizio 488 and analyzed with Image Cell software (Mauna Kea Technologies™, France).

### Immunohistochemistry

Freshly dissected mouse femurs were fixed in 4% neutral-buffered paraformaldehyde for 24 hours. Fixed specimens were decalcified, embedded in paraffin, sectioned at 4μm, mounted on glass slides, and leukemic cells detected by immunochemistry as described below. Briefly, endogenous peroxidase activity was removed with 5% H_2_O_2_ in PBS for 15 minutes. CD45 protein was detected using a 1:1000 dilution of rabbit anti-human CD45 (Abcam, Cambridge, England) for 45 minutes at room temperature. After washing with PBS, HRP secondary anti-rabbit immunoglobulin antibody (ImmPRESS™ kit, Vector, Philadelphia, USA) was added for 30 minutes. Finally, staining was revealed using diaminobenzidine (DAB) and sections counterstained using hematoxylin. Control experiments were performed with purified rabbit IgG. Slides were observed using Leica DFC310Fx microscope.

### DNA extraction and genomic DNA array analysis

DNA was extracted from patient samples using the QIAmp DNA mini kit (Qiagen, Hilden, Germany) according to the manufacturer's instructions. Nucleic acid quantity was assessed (ND-1000; NanoDrop) and genomic DNAs analysed [11]. Briefly, DNA from CD7^+^/CD34^+/−^ cell fractions at diagnosis and after xenograft was analyzed by high-density array comparative genomic hybridization (CGH) technologies using the 1 × 1M Microarray SurePrint G3 Catalog (Agilent Technologies) or 4 × 180K according to the manufacturers’ recommendations. Analyses were performed using the Genomic Workbench software (Agilent Technologies).

### PCR for IKZF1 intragenic deletions

IKZF1 deleted cells were detected in CD7^+^/CD34^+/−^ fractions from T-ALL3 sample at diagnosis and after xenografts and quantified as previously described [17].

### Statistical analysis

For leukemia infiltration into bone marrow, statistical significance of comparisons was determined using the 2-tailed Mann and Whitney non-parametric test using GraphPad Prism (GraphPad Software, Inc. La Jolla, CA, USA). Statistics are evaluated in so far as the number of total or positive mice allows. *, P < 0.05; **, P < 0.01; and ***, P < 0.001 were considered statistically significant. For leukemia initiating cells frequency, statistical analyses were performed using a one-way ANOVA test provided in ELDA software.

## SUPPLEMENTARY MATERIALS AND METHODS


