# Supplementary material for: Speed of leukemia development and genetic diversity in xenograft models of T cell acute lymphoblastic leukemia
Source: Oncotarget. 2016 May 12;7(27):41599–611. doi: 10.18632/oncotarget.9313 (PMC5173081; doi:10.18632/oncotarget.9313)
Supplement: Supplementary file 1 [file oncotarget-07-41599-s001.pdf]

Speed of leukemia development and genetic diversity in xenograft models of T cell acute lymphoblastic leukemia

SUPPLEMENTARY MATERIALS AND METHODS

Targeted exome sequencing

Library preparation, exome capture, sequencing and data analysis have been done by IntegraGen SA (Evry, France).

Genomic DNA is captured using Agilent in-solution enrichment methodology (SureSelect XT Clinical Reasearch Exome, Agilent) with their biotinylated oligonucleotides probes library (SureSelect XT Clinical Reasearch Exome - 54 Mb, Agilent), followed by paired-end 75 bases massively parallel sequencing on Illumina HiSeq4000. For detailed explanations of the process, see Gnirke publication in Nature Biotechnology [1].

Sequence capture, enrichment and elution are performed according to manufacturer's instruction and protocols (SureSelect, Agilent) without modification except for library preparation performed with NEBNext® Ultra kit (New England Biolabs®). For library preparation 600 ng of each genomic DNA are fragmented by sonication and purified to yield fragments of 150-200 bp. Paired-end adaptor oligonucleotides from the NEB kit are ligated on repaired A-tailed fragments, then purified and enriched by 8 PCR cycles. 1200ng of these purified Libraries are then hybridized to the SureSelect oligo probe capture library for 72 hr. After hybridization, washing, and elution, the eluted fraction is PCR-amplified with 9 cycles, purified and quantified by qPCR to obtain sufficient DNA template for downstream applications. Each eluted-enriched DNA sample is then sequenced on an Illumina HiSeq4000 as paired-end 75b reads. Image analysis and base calling is performed using Illumina Real Time Analysis (2.7.3) with default parameters.

Bioinformatics

Base calling is performed using the Real-Time Analysis software sequence pipeline (2.7.3) with default parameters. Sequence reads were mapped to the human genome build (hg19 / GRCh37) using Elandv2e (Illumina, CASAVA1.8.2) allowing multiseed and gapped alignments. The duplicated reads (e.g. paired-end reads in which the insert DNA molecule showed identical start and end locations in the human genome) are removed.

CASAVA1.8.2 is used to call single-nucleotide variants (SNVs) and short insertions/deletions (max. size is 300nt), taking into account all reads per position.

We used an in-house IntegraGen SA (Evry, France) algorithm which compares normal and tumor genotypes from exome sequencing data to determine the somatic nature of the variation. A somatic score is calculated for each variant ranging from 1 to 30, a score of 30 translating the highest confidence index. This score takes into account the frequencies and counts of mutated allele in both samples to minimize false positive variations. Finally, variants displaying mutated reads in the constitutional sample above 5 percent are considered as germline or false positive and eliminated to the somatic tab. The somatic variant caller handles indels similarly, analyzing the number of alignments covering a given position that include a particular indel (the variant count) versus the overall coverage at that position. For SNV analysis, samples with a SNV quality (Qsnv)<20 and somatic score<10 were eliminated. Remaining hazardous (Qsnv<20 and somatic score≥10) and non-hazardous (Qsnv>20 and somatic score≥5) somatic insertions/deletions and SNV were checked using IGV2.3.72 Software.

Variants annotation takes into account data available in dbSNP (dbSNP144), the 1000 Genomes Project (phase1_release_v3.20101123), the Exome Variant Server (ESP6500SI-V2-SSA137), and the Exome Aggregation Consortium (ExAC r3.0) and from an in-house database (201 exomes whole exomes for SNVs and 130 exomes whole exomes for indels). Functional consequences of variants on genes, transcripts, and protein sequence, as well as regulatory regions, are predicted by Variant Effect Predictor (VEP release 83) (stop, splicing, missense, synonymous…), as well as by location of the variants (e.g. upstream of a transcript, in coding sequence, in non-coding RNA, in regulatory regions). Regarding missense changes, two bioinformatics predictions for pathogenicity were available SIFT (sift5.2.2), PolyPhen (2.2.2). Other information like quality score, homozygote/heterozygote status, count of variant allele reads, mutation type (somatic or germline) and somatics score, the presence of the variant in the COSMIC database (version71) are reported.

REFERENCES

1. Gnirke A, Melnikov A, Maguire J, Rogov P, LeProust EM, Brockman W, Fennell T, Giannoukos G, Fisher S, Russ C, Gabriel S, Jaffe DB, Lander ES, et al. Solution hybrid selection with ultra-long oligonucleotides for massively parallel targeted sequencing. Nat Biotechnol. 2009; 27:182-9. doi: 10.1038/nbt.1523.

SUPPLEMENTARY FIGURES AND TABLES

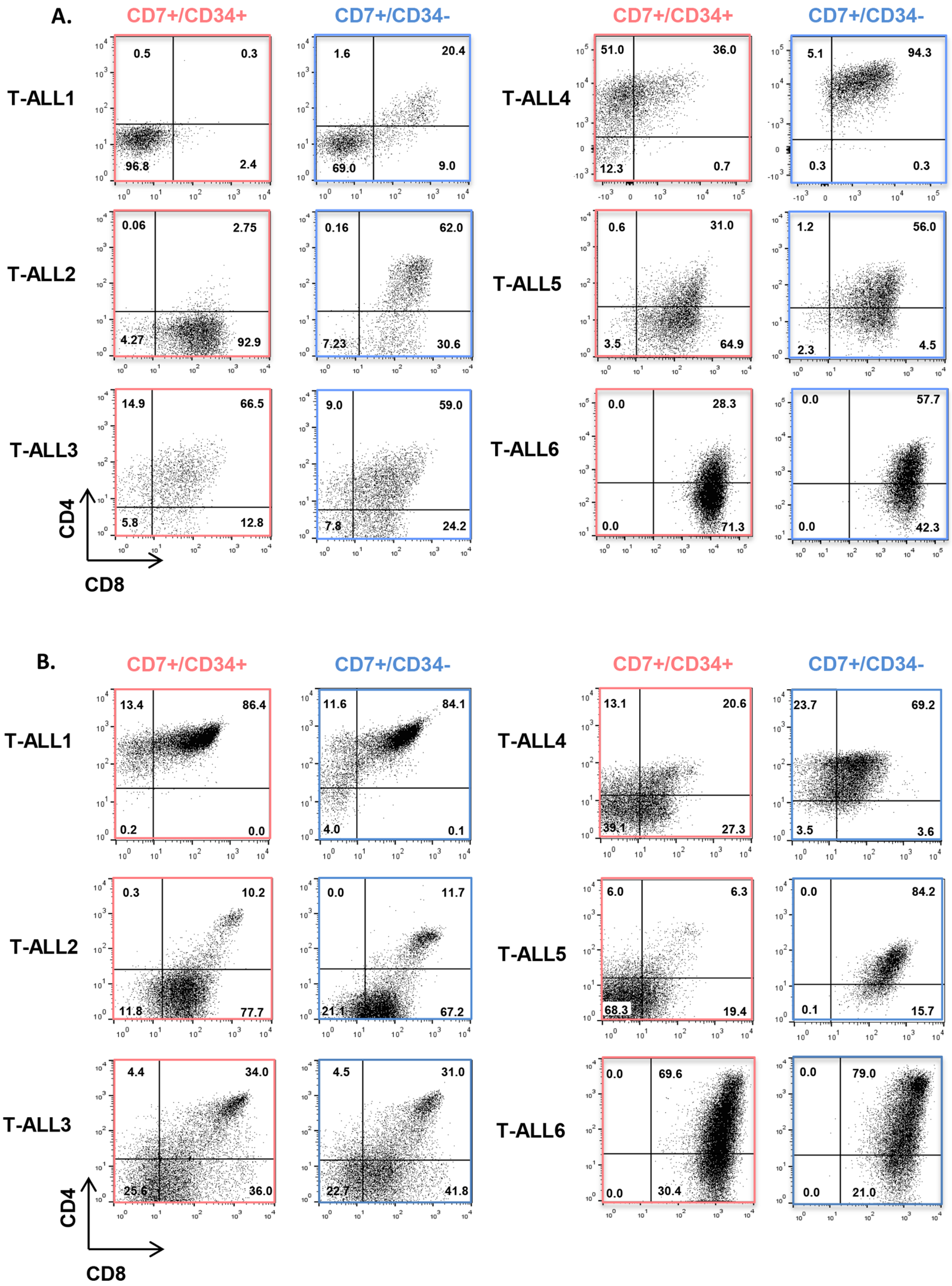

Supplementary Figure S1: Phenotype of CD7+/CD34+ and CD7+/CD34- T-ALL cells at diagnosis A. and after xenograft B. CD34+/CD7+ or CD34-/CD7+ cells were sorted (see in Figure 1 and 3 for sorting purity) and transplanted into NSG mice. Shown is CD4 and CD8 expression in CD34+/- CD7+ from T-ALL at diagnosis (A) and from xenografted T-ALL recovered from BM of mice (B). Dot plots in B are from representative mice.

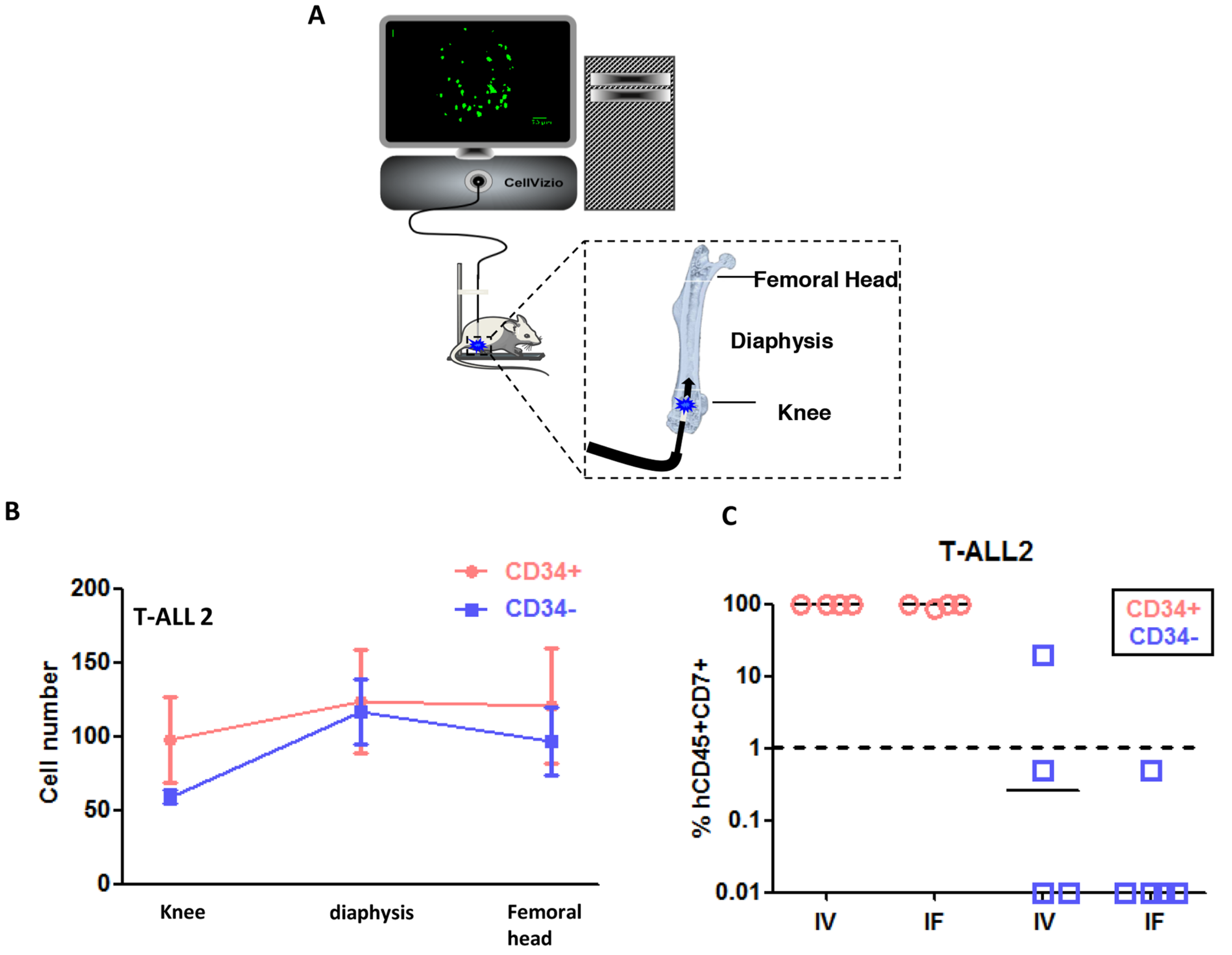

Supplementary Figure S2: Follow up of leukemia infiltration into bone marrow early after transplantation. A. CD34+/CD7+ or CD34-/CD7+ T-ALL2 (5x10^4/mouse) cells were sorted, labelled with CFSE and transplanted into NSG mice. Migration and localization were measured 72 hours later using a cell vizio apparatus. **B.** Number of cells counted at 72 hours. **C.** $CD7^+/CD34^+$ or $CD7^+/CD34^-$ T-ALL2 (5x10^3/mouse) cells were injected by intravenous (IV) or intrafemoral (IF) routes in NSG mice. Percent of $hCD45^+/CD7^+$ leukemic cells was evaluated in BM 14 weeks after transplant of T-ALL2. Statistics were determined using the 2-tailed Mann and Whitney test.

A

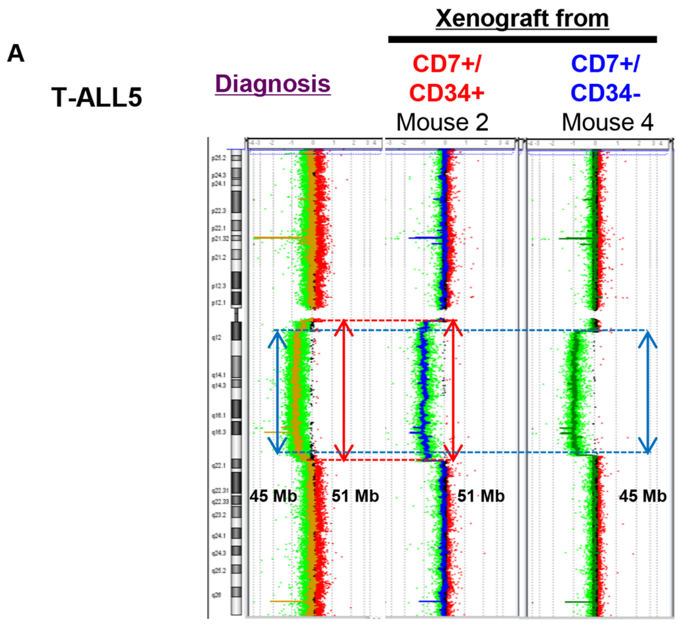

B

T-ALL5

Diagnosis

100% del 9p21.3 (CDKN2A/B)
100% del 14q11.2 (TCRA/D)
50% del 6q12-21 51Mb
50% del 6q12-21 45Mb

Xenograft resulting from the transplantation of

Cell fraction injected	CD7+/ CD34+			CD7+/ CD34-
Cell number/mouse	1x10^5		5x10^4	1x10^5
mouse	Mouse 1	Mouse 2	Mouse 3	Mouse 4
Genetic changes	100% del 9p21.3 (CDKN2A/B) 100% del 14q11.2 (TCRA/D)			
	80-100% del 6q12-21 - 51Mb			100% del 6q12-21 45Mb
	-	20% trisomy 16 20% trisomy 21	20 % del 6q12-21 – 43.2Mb 4,5,5,der(4) t(4;5) (p;p)	-

Supplementary Figure S3: Array-CGH analyses of leukemia samples at T-ALL5 patient's diagnosis or recovered from mice. Array-CGH analysis of leukemic cells at diagnosis and total cells recovered following xenograft of CD7$^+$/CD34$^+$ and CD7$^+$/CD34$^-$ cells. **A.** Array-CGH plots of 6q deletions discriminating CD7$^+$/CD34$^+$ and CD7$^+$/CD34$^-$-derived leukemias following xenograft, compared to diagnosis. **B.** Results obtained from CGH-array analyses. (*Continued*)

C

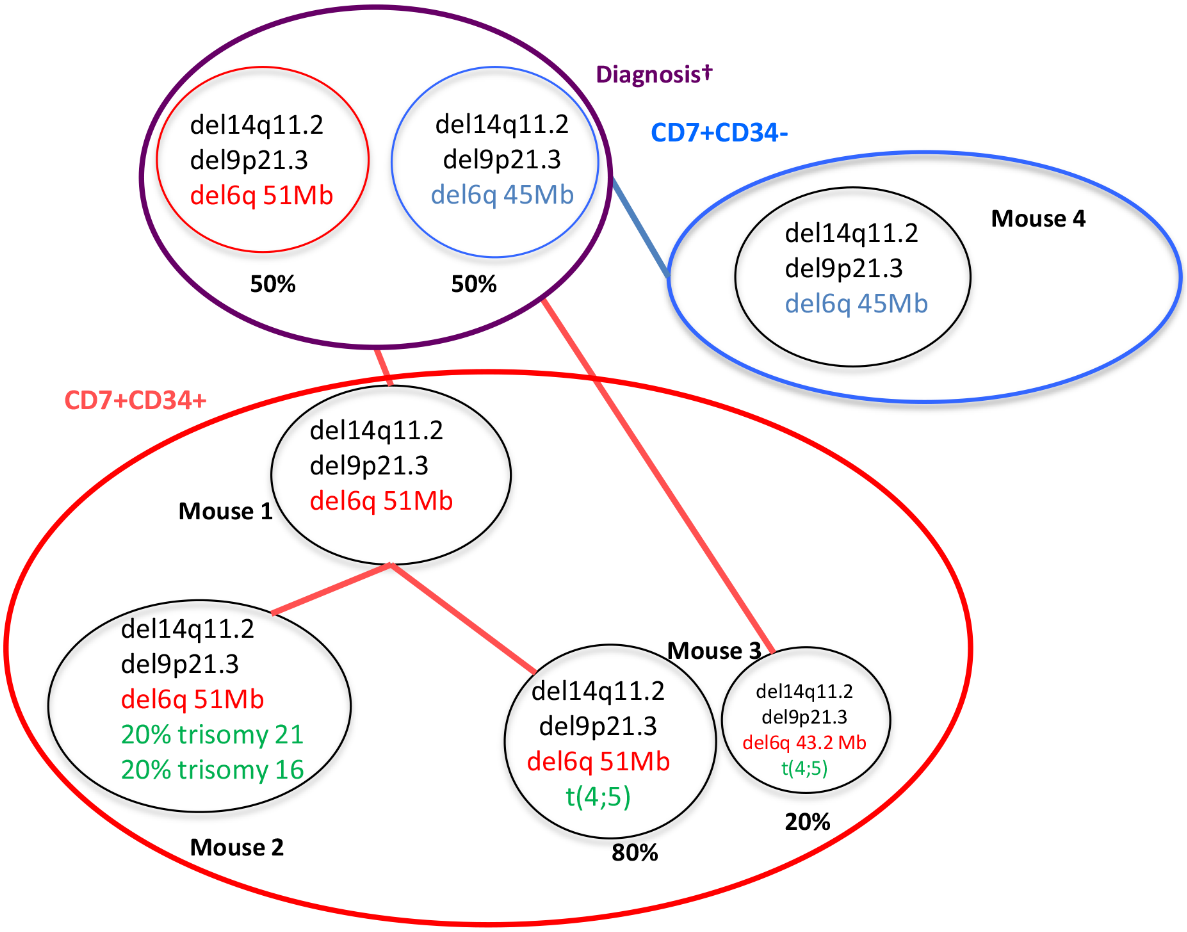

Supplementary Figure S3: (*Continued*) Array-CGH analyses of leukemia samples at T-ALL5 patient's diagnosis or recovered from mice. C. Hypothetical phylogeny of genetic evolution based on array-CGH results obtained at diagnosis and for $CD7^+/CD34^+$- and $CD7^+/CD34^-$ -derived leukemia. Percentages of cells with genetic alterations were evaluated based on log2 ratio values.† are shown only genomic alteration in cells detected by array-CGH. Red and blue circles respectively indicate genomic alterations in $CD7^+/CD34^+$ and $CD7^+/CD34^-$ cells (at diagnosis or derived from the xenograft). Purple and black circles shown genomic alterations at diagnosis and after xenograft in NSG mice respectively. The alterations in red and blue are representatives of $CD7^+/CD34^+$ and $CD7^+/CD34^-$ populations respectively. The alterations in green are probably presents in distinct subfractions without clear distribution within cell populations.

Supplementary Table S1: Biological characteristics of human T-ALL samples used

T-ALL samples	Sex	EGIL maturation stage	White Blood cell (G/L)	NOTCH1 mutations	Oncogenic transcripts	Clinical follow-up	% of CD34+ cells at diagnosis
T-ALL1/M105	M	Cortical	129	HD	TAL1/LMO2	CR1, allograft, CR2	17
T-ALL2/M121	F	Pre-T	108	WT	LMO2	CR1	90
T-ALL3/M106	F	Mature	743	WT	SIL/TAL deletion	died 2 days after diagnosis	30
T-ALL4/M112	F	Mature	109	WT	TLX3	CR1	38
T-ALL5/M22	M	Cortical	375	WT	NEG	CR1	60
T-ALL6/M145	M	Cortical	220	HD	NEG	Too short after diagnosis	6

M# refers to T-ALL as previously described (Armstrong, Blood, 2009; Gerby, Leukemia, 2011). CR= complete remission. The follow-up period was 2 years.

Supplementary Table S2: Genetic modifications observed at diagnosis and after xenograft of human $CD7^+/CD34^+$ and $CD7^+/CD34^-$ cells from T-ALL1

T-ALL 1 sample			DIAGNOSIS		XENOGRAFT			
Chr	Targeted genes	Genetic changes	CD7+/CD34+	CD7+/CD34-	#5x10^3 cells		5x10^2 cells	
					†CD7+/CD34+	CD7+/CD34-	CD7+/CD34+	CD7+/CD34-
					m1	m5	m1	m5
1q22	FCRL4	gain	yes	yes	yes	yes	yes	yes
7p14.1	TARP (TCRγ)	deletion	yes	yes	yes	yes	yes	yes
7q34	PRSS1/PRSS2/EPHB6/TRY6/TRPV5/TRPV6/KEL/OR9A2/07orf34 (TCRβ)	deletion	yes	yes	yes	yes	yes	yes
9p21.3	CDKN2A/BC9orf53	deletion	yes	yes	yes	yes	yes	yes
13q14.2	RCBTB2/P2RY5/RB1	deletion	yes	yes	yes	yes	yes	yes
14q22.1	LOC176913	deletion	yes	yes	yes	yes	yes	yes

Sorted cells from primary samples (DIAGNOSIS) and total human T-ALL cells recovered from mouse (m) BM (XENOGRAFT) were analysed using array-CGH. # number of cells transplanted per mouse. † Population of sorted cells transplanted into NSG mice.

Supplementary Table S3: Genetic modifications observed at diagnosis and after xenograft of human CD7$^+$/CD34$^+$ and CD7$^+$/CD34$^-$ cells from T-ALL2

T-ALL 2 sample			DIAGNOSIS		XENOGRAFT							
Chr	Targeted genes	Genetic changes	CD7+/ CD34+	CD7+/ CD34-	5x10^4 cells #				5x10^3 cells			
					†CD7+/CD34+		CD7+/CD34-		CD7+/CD34+		CD7+/CD34-	
					m1	m4	m7	m8	m1	m2	m5	m6
7p14.1	TARP (TCRγ)	deletion	yes	yes	yes	yes	yes	yes	yes	yes	yes	yes
7q34	PRSS1	deletion	yes	yes	yes	yes	yes	yes	yes	yes	yes	yes
7q34	TRY6 (TCRβ)	deletion	yes	yes	yes	yes	yes	yes	yes	yes	yes	yes

Sorted cells from primary samples (DIAGNOSIS) and total human T-ALL cells recovered from mouse (m) BM (XENOGRAFT) were analysed using array-CGH. # number of cells transplanted per mouse. † Population of sorted cells transplanted into NSG mice.

Supplementary Table S4: Genetic modifications observed at diagnosis and after xenograft of human CD7$^+$/CD34$^+$ and CD7$^+$/CD34$^-$ cells from T-ALL3

T-ALL3			DIAGNOSIS	XENOGRAFT			
Chr	Genes	Genomic difference		5x10^1 cells #		5x10^2 cells	
				†CD7+/CD34+		CD7+/CD34-	
				Mouse 1	Mouse 3	Mouse 10	Mouse 11
1p33	TAL1/STIL	deletion	yes	yes	yes	yes	yes
7p14.1	TARP	deletion	yes	yes	yes	yes	yes
7q34	PRSS1/TRY6/PRSS2	deletion	yes	yes	yes	yes	yes
9p21.3	C9orf53/CDKN2A	deletion	yes	yes	yes	yes	yes
10q23.31	Many genes (PTEN)	deletion	yes	yes	yes	yes	yes
12q23.1	Many genes	deletion	yes	yes	yes	yes	yes
12q24.32-24.33	Many genes	deletion	yes	yes	yes	yes	yes
13q14.2-q21.31	Many genes (RB1)	deletion	yes	yes	yes	yes	yes

Sorted cells from primary samples (DIAGNOSIS) and total human T-ALL cells recovered from mouse (m) BM (XENOGRAFT) were analysed using array-CGH. # number of cells transplanted per mouse. † Population of sorted cells transplanted into NSG mice.

Supplementary Table S5: Genetic modifications observed at diagnosis and after xenograft of human $CD7^{+}/CD34^{+}$ and $CD7^{+}/CD34^{-}$ cells from T-ALL6

T-ALL6			DIAGNOSIS	XENOGRAFT			
				5x10^5 cells #			
				†CD7+/CD34+		CD7+/CD34-	
**Chr**	**Genes**	**Genomic difference**		**Mouse 3**	**Mouse 4**	**Mouse 1**	**Mouse 3**
1p36.13	NBPF1/ CROCCL1/ MST1P2/ ESPNP/ MST1P9/ CROCC	deletion	yes	yes	yes	yes	yes
6q23.3	MYB, AHI1	gain	yes	yes	yes	yes	yes
7p14.1	TARP	deletion	yes	yes	yes	yes	yes
10q11.22	Many genes	deletion	yes	yes	yes	yes	yes
17p13.3-p13.1	Many genes	deletion	yes	yes	yes	yes	yes
21q11.2-q22.3	Many genes	gain	yes	yes	yes	yes	yes

Sorted cells from primary samples (DIAGNOSIS) and total human T-ALL cells recovered from mouse (m) BM (XENOGRAFT) were analysed using array-CGH. # number of cells transplanted per mouse. † Population of sorted cells transplanted into NSG mice.
